# Antioxidant and antiproliferative activities of *Abrus precatorius* leaf extracts - an *in vitro* study

**DOI:** 10.1186/1472-6882-13-53

**Published:** 2013-03-02

**Authors:** Mir Z Gul, Farhan Ahmad, Anand K Kondapi, Insaf A Qureshi, Irfan A Ghazi

**Affiliations:** 1 Department of Plant Sciences, School of Life Sciences, University of Hyderabad, Prof. C. R. Rao Road, Gachibowli, Hyderabad 500 046, India; 2 Department of Biotechnology, School of Life Sciences, University of Hyderabad, Prof. C. R. Rao Road, Gachibowli, Hyderabad, 500 046, India

## Abstract

**Background:**

The use of traditional medicine at the primary health care level is widespread and plant-based treatments are being recommended for curing various diseases by traditional medical practitioners all over the world. The phytochemicals present in the fruits, vegetables and medicinal plants are getting attention day-by-day for their active role in the prevention of several human diseases. *Abrus precatorius* is a widely distributed tropical medicinal plant with several therapeutic properties. Therefore in the present study, *A. precatorius* leaf extracts were examined for their antioxidant and cytotoxic properties *in vitro* in order to discover resources for new lead structures or to improve the traditional medicine.

**Methods:**

In this study, antioxidant and antiproliferative properties of the different leaf extracts (hexane, ethyl acetate, ethanol and water) from *A. precatorius* were investigated along with the quantification of the polyphenol and flavonoid contents. The ability of deactivating free radicals was extensively investigated with *in vitro* biochemical methods like DPPH^•^, ^•^OH, NO, SO_2-_ scavenging assays and inhibition capability of Fe(II)-induced lipid peroxidation. Furthermore, antiproliferative activities using different human cancer cell lines and primary cell line was carried out by MTT method.

**Results:**

Total phenolic content and total flavonoid content of the extracts were found in the range of 1.65 ± 0.22 to 25.48 ± 0.62 GAE mg/g dw and 6.20 ± 0.41 to 17.16 ± 1.04 QE mg/g dw respectively. The experimental results further revealed that *A. precatorius* extracts showed strong antiradical properties, capable to chelate Fe^2+^ and possess good inhibition ability of lipid peroxidation. In addition, as a first step towards the identification of phytoconstituents endowed with potent chemopreventive activities, we evaluated the inhibitory effects of *A. precatorius* extracts on the proliferation of four different human tumour cell lines such as human colon adenocarcinoma cells (Colo-205), human retinoblastoma cancer cells (Y79), human hepatocellular carcinoma cells (HepG2) and Leukemia cells (SupT1). Ethanol extract (APA) and ethyl acetate extract (APE) of *A. precatorius* had apparent capabilities of inhibiting the survival of tested human cancer cell lines. Moreover, it was observed that the *A. precatorius* extracts did not inhibit the growth of mice peritoneal macrophages, thus confirming that plants extracts are selective against the cancer cell lines.

**Conclusion:**

This work provides a scientific support for the high antioxidant and antiproliferative activity of this plant and thus it may find potential applications in the treatment of the diseases caused by ROS. Further studies are needed to confirm *in vivo* anti-tumorgenicity and subsequent chemical characterization of the active molecule(s).

## Background

The human body possesses numerous antioxidant defences and repair mechanisms against oxidative stress. However, these mechanisms are insufficient to prevent the damage entirely as production of reactive oxygen species (ROS) is certain to play multiple important roles in tissue damage and loss of function in a number of tissues and organs [1]. Free radicals and ROS have been implicated as endogenous initiators in the etiology of cancer and several other degenerative or pathologic processes of various serious diseases, as well as in aging processes [2]. Oxidative damage to DNA is considered a critical step in cancer development [3]. Over the past decade or so, numerous experimental and epidemiological studies have shown that a wide variety of phytochemicals such as phenolics, flavonoids, isoflavone, flavones, anthocyanins, catechin, isocatechin and carotenoids are able to prevent or slow down oxidative stress-induced damage leading to carcinogenesis by upsetting the molecular events in the initiation, promotion or progression conditions. Recent studies demonstrated that the high dietary intake of fruits and vegetables could be associated with lower cancer prevalence in humans [4-7]. Natural products mainly from plant kingdom offer a wide range of biologically active compounds that act as natural antioxidants with recognized potential in drug discovery and development [8]. Great interest is currently being paid to natural products for their interesting anticancer activities. High percentages (~ 60%) of all the drugs applied in the treatment and/or prevention of cancer are from natural products and their derivatives, of which higher plants contribute around 25%. Approximately 60% of drugs approved for cancer treatment are of natural origin [9,10]. This has elicited the pursuit of effective antioxidant and anticancer agents from various sources particularly medicinal and edible plants [11]. Investigations on natural products have regained prominence in the recent past with increasing understanding of their biological significance such as antioxidant, radical scavenging, antiproliferative activities and increasing recognition of the origin and function of their structural diversity [12-15].

*Abrus precatorius* L. (Fabaceae) is a vine originally native to India that is now commonly found throughout the tropical and subtropical parts of the world [16]. It grows best in fairly dry regions at low elevations. Leaves, roots and seeds are used as a medicament in traditional system of Indian medicine for antihelminthic, antidiarrhoeal, antiemetic and inhibits intestinal motility. Researchers have reported that seeds are used for the treatment of diabetes and chronic nephritis [17]. The leaves of *A. precatorius* are sweeter [18,19] and as equivalent in sweetness potency to sucrose [20]. In West Tropical Africa, *A. precatorius* leaves have been employed to sweeten foods and certain medicines used for stomach complaints, to treat fevers, cough and cold (used as decoction). The leaves are casually chewed and the vine sometimes sold as a masticatory in Curacao [21,22]. The plant is also traditionally used to treat tetanus, and to prevent rabies. Though considerable work has been done on the seeds for different activities, however, scientific information on antioxidant and antiproliferative properties of leaves of this plant is still not available or rather scarce. Thus, we evaluated the abilities of leaf extracts of *A. precatorius* to function as an antioxidant agent using *in vitro* assays. Additionally, the ability of the leaf extracts to inhibit proliferation of various cancer cell lines was investigated. Since elimination of cancer in early stages is an integral part of chemoprevention, measuring antiproliferative properties against cancer cells provide useful insight on the chemo-protective potential of natural extracts. Thus, the objective of this study was to examine the efficacy of *A. precatorius* as an antioxidant as well as its inhibitory effect on human cancer cell proliferation.

## Methods

### Chemicals

The analytical grade chemicals were purchased from Hi-Media and Merck, India. Standard drugs were purchased from Sigma-Aldrich chemicals co. (Germany); RPMI-1640, DMEM and foetal bovine serum (FBS) from Gibco (USA).

### Plant material

*Abrus precatorius* leaves were kindly provided by Central Research Institute of Unani Medicine, Hyderabad. A voucher specimen (UoH/VS/AP-2) has been preserved in our laboratory for future reference.

### Preparation of extracts

The air-dried leaves of the plant were powdered with a mechanical grinder to obtain a coarse powder, which was then subjected to successive extraction in a soxhlet apparatus using hexane, ethyl acetate, ethanol and water. Each time before extracting with the next solvent, the material was dried in hot air oven at 40°C. Extracts were then filtered through a Whatman No.1 paper filter and concentrated to the dry mass with the aid of rotary evaporator. The extraction process was repeated three times at different time periods. It was observed that there was no significant difference in the percentage yield and content of phyto-constituents that are believed to play an important role in biological activities. The yield of each extract was measured and residues were stored in dark glass tubes for further analysis. The different extracts were designated as APH (for hexane extract), APE (for ethyl acetate extract), APA (for ethanol extract) and APW (for water extract). The dried extracts were dissolved in dimethyl sulfoxide (DMSO) as 20 mg/mL and diluted with phosphate-buffered saline (PBS, pH 7.4) to give final concentrations.

### Determination of phytoconstituents

#### Determination of total phenols

Total phenolics were determined using Folin-Ciocalteu reagent as described by Yang *et al.*[23] with minor modifications. Total phenolic assay was conducted by mixing 2.7 mL of deionised water, 0.01 mL of extracts, 0.3 mL 20% Na_2_CO_3_ and 0.10 mL Folin-Ciocalteu reagent. Absorbance of mixture was measured at 725 nm. A standard curve was prepared with gallic acid (r^2^ = 0.9454) and final results were given as mg gallic acid equivalents (GAE) g dw.

#### Determination of total flavonoids

The total flavonoids was measured by addition of aluminium chloride reagent to the solution containing the extract using the method of Barrera et al. [24]. Briefly, 10 μL of plant extracts of known concentrations (20 mg/mL) were diluted with 0.5 mL of double distilled water. To this mixture, 30 μL of 5% sodium nitrite (NaNO_2_) and 60 μL of 10% aluminium chloride were added and incubated at room temperature for 10 min. After incubation, 350 μL of 1 M NaOH was added and total volume was made up to 1 mL with distilled water. Finally, absorbance was measured against the prepared blank at 510 nm and results were given as quercetin equivalents (mg QE)/g of dw. Standard curve was prepared with known concentrations of quercetin (r^2^ = 0.955).

### Antioxidant ability assays

#### Phosphomolybdenum assay

The total antioxidant activity of extracts was evaluated by green phosphomolybdenum complex according to the method of Prieto *et al.*[25]. An aliquot of 10 μL of sample solution was mixed with 1 mL of reagent solution (0.6 M sulphuric acid, 28 mM sodium phosphate and 4 mM ammonium molybdate) in micro centrifuge tube. Tubes were incubated in a dry thermal bath at 95°C for 90 min. After cooling, the absorbance of the mixture was measured at 695 nm against a blank. Ascorbic acid have been used (r^2^ = 0.964) for reference and the reducing capacities of the analyzed extracts were expressed as mg of ascorbic acid equivalents (mg AAE)/g of dw.

#### Ferric-reducing/antioxidant power (FRAP) assay

The Fe^3+^ reducing power of the extracts was determined by the method of Oyaizu [26] with slight modifications. Briefly, extracts and standard (ascorbic acid) in 1 mL of appropriate solvents were mixed with 2.5 mL of phosphate buffer (0.2 M, pH 6.6) and 2.5 mL of potassium ferricyanide (1%), and then mixture was incubated at 50°C for 30 min. Afterwards, 2.5 mL of trichloroacetic acid (10%) was added to the mixture, which was then centrifuged at 5000 rpm for 10 min. Finally, 2.5 mL of the upper layer solution was mixed with 2.5 mL of distilled water and 0.1 mL of FeCl_3_ (0.1%). The absorbance was measured at 700 nm and the reducing power of the extracts was presented as mg AAE/g of dw.

#### DPPH^•^ radical scavenging activity

The DPPH free radical scavenging activity of leaf extracts of *A. precatorius* was measured in term of hydrogen donating or radical scavenging ability using the stable radical DPPH [27]. Briefly, 0.004% w/v of DPPH radical solution was prepared in methanol and then 900 μL of this solution was mixed with 100 μL of extract solution containing 40–400 μg/mL of dried extract. The absorbance was measured at 517 nm after 30 min of incubation. Methanol (95%), DPPH solution and ascorbic acid were used as blank, control and reference respectively.

#### Hydroxyl radical scavenging activity

The ability of the extracts to inhibit site-specific hydroxyl radical-mediated peroxidation was carried out according to the method given by Hinneburg *et al*. [28] with some modifications. Briefly, the mixture containing FeCl_3_ (10 mM), ascorbic acid (1 mM), H_2_O_2_ (10 mM), deoxyribose (28 mM) and different concentrations of test samples (40–400 μg/mL) in 500 μL phosphate buffered saline (PBS, 20 mM, pH 7.4) was incubated for 30 min at 37°C. After adding 1 mL of trichloroacetic acid (10%, w/v) and 1 mL thiobarbituric acid (2.8% w/v; in 25 mM NaOH), the reaction mixture was boiled for 15 min. The extent of oxidation was estimated at 532 nm and the scavenging activity of test sample was expressed as the percentage inhibition of the deoxyribose degradation to malondialdehyde. Ascorbic acid was used as the positive control.

#### Hydrogen peroxide scavenging assay

The ability of plant extracts to scavenge hydrogen peroxide was determined according to Long *et al*. [29]. A 40 mM of H_2_O_2_ solution was mixed with different concentrations of plant extracts (20–200 μg/mL) and incubated for 3.5 h at room temperature. After incubation, 90 μL of the H_2_O_2_-sample solution was mixed with 10 μL of HPLC-grade methanol and 0.9 mL of FOX reagent was added (prepared by mixing 9 volumes of 4.4 mM BHT in HPLC-grade methanol with 1 volume of 1 mM xylenol orange and 2.56 mM ammonium ferrous sulfate in 0.25 M H_2_SO_4_). The reaction mixture was vortexed and then incubated at room temperature for 30 min. The absorbance of ferric-xylenol orange complex was measured at 560 nm. Ascorbic acid was used as the reference compound.

#### Nitric oxide scavenging activity

The free radical scavenging potential of *A. precatorius* was further substantiated by scavenging of nitric oxide radical assayed by sodium nitroprusside method [30]. The reaction solution (50 μL) containing 10 mM sodium nitroprusside in PBS (pH 7.0) was mixed with different concentration (40–400 μg/mL) of sample extracts, followed by incubation at 37°C for 20 min under light. After incubation, the samples were mixed with 300 μL of Griess reagent (1% sulfanilamide, 2% H_3_PO_4_)_._ The samples were again incubated for 30 min at room temperature under light conditions followed by the addition of 0.1% N-(1-naphthyl) ethylenediamine dihydrochloride. The absorbance was recorded at 546 nm and the results were expressed as per cent of scavenged nitric oxide with respect to the negative control without addition of any antioxidant. Ascorbic acid was used as a positive control.

#### Superoxide radicals scavenging activity

The scavenging activity of the plant extracts towards superoxide anion radicals was measured by the nitro-blue tetrazolium (NBT) reduction method [31] with minor modifications. Superoxide anions were generated in a non-enzymatic phenazine methosulfate nicotinamide adenine dinucleotide (PMS-NADH) system through the reaction of PMS, NADH and oxygen. It was assayed by the reduction of nitroblue tetrazolium. In the experiment, the superoxide anion was generated in 2 mL of phosphate buffer (100 mM, pH 7.4) containing 500 μL of 156 μM nitroblue tetrazolium (NBT solution), 500 μL of 468 μM nicotinamide adenine dinucleotide (NADH) solution and 300 μL of different concentrations (40–400 μg/mL) of extracts. DMSO and L-ascorbic acid were used as solvent and positive control respectively. The reaction was initiated by adding 100 μL of 60 μM phenazine methosulfate (PMS) to the mixture. After 5 min of incubation at room temperature, the absorbance was measured at 560 nm against blank. Decreased absorbance of the reaction mixture indicated increased superoxide anion scavenging activity.

#### Inhibition of lipid peroxidation assay

Fe^2+^ induced lipid peroxidation is one of the established system for assessing antioxidant action of different plant extracts. A modified thiobarbituric acid-reactive species (TBARS) assay [32] was used to measure the lipid peroxide formed using rat liver homogenate as lipid rich media. Malondialdehyde (MDA), a secondary end product of the oxidation of polyunsaturated fatty acids, reacts with two molecules of TBA yielding a pinkish red chromogen. Healthy albino rats of the wister strain (250 grams) were sacrificed (procedure described was reviewed and approved by the University of Hyderabad, School of Life Sciences’ animal ethics committee) and liver was perfused with 0.15 M KCl, homogenate was centrifuged at 800 g for 15 min at 4°C and the supernatant was used for thiobarbituric acid assay. The extracts of *A. precatorius* at different concentrations (40–400 μg/mL) were mixed with the liver microsome preparation and incubated at room temperature for 10 min. Then, 50 μL Fenton’s reagent (10 mM FeCl_3_; 10 μL of 2.5 mM H_2_O_2_; 0.1 M L-ascorbic acid) in phosphate buffer (0.2 M, pH 7.4) were added, and the volume was made to 1 mL. The tubes were then incubated for 30–45 min at 37°C to induce lipid peroxidation. Thereafter, 2 mL of ice-cold HCl (0.25 N) containing 15% trichloroacetic acid, 0.5% thiobarbituric acid and 0.5% butylated hydroxytoluene (BHT) were added in each sample followed by heating at 100°C for 15 min. The reaction mixture was put in an ice bath for 10 min. The mixture was centrifuged at 1000 rpm for 10 min and the extent of lipid peroxidation was subsequently monitored by formation of thiobarbituric acid reactive substances (TBARS) as pink chromogen in presence or absence of extracts and standard (L-ascorbic acid). The absorbance of the supernatant was measured spectrophotometrically at 532 nm and decline in formation of pink chromogen in pre-treated reactions was considered as inhibition of lipid peroxidation.

#### Anti-proliferative activity

A panel of four human cell lines namely, (a): human colon adenocarcinoma cells - Colo-205, (b): human retinoblastoma cancer cells - Y79, (c): human hepatocellular carcinoma cells - HepG2 and (d): Leukaemia cells - SupT1 were used to study antiproliferative activity. The cell lines were obtained from National Centre for Cell Sciences (NCCS), Pune, India. The cell lines HepG2 & Colo-205 were cultured in Dulbecco’s modified Eagle’s medium (DMEM) and Y79 & SupT1 in RPMI 1640 containing 10% (v/v) FBS, 100 units/mL penicillin and 100 μg/mL streptomycin. Cells were maintained in a humidified incubator with 5% CO_2_ for 24 h at 37°C and seeded onto 75 cm^2^ culture flasks. *In vitro* response to extracts and standard drug was evaluated by means of a growth inhibition using the MTT assay [33]. The cells were seeded at a density of ~5 × 10^3^ per well using a brief trypsinization. Furthermore, Doxorubicin and sample extracts (25–200 μg/mL) dissolved in dimethylsulfoxide (DMSO; not exceeding the concentration of 2%), and further diluted in cell culture medium were added into a 96-well plate. After 48 h of incubation, 20 μL of MTT reagent (5 mg/mL) were added and mixtures were reincubated for 4 h. Finally the absorbance of formazan was measured at 550 nm. Doxorubicin was also assayed as a positive control at the concentration of 0.5–10 μg/mL. The resulting growth data represents the net outcome of cell proliferation and cell death. The cell viability (%) was obtained by comparing the absorbance between the samples and a negative control.

#### Assessment of extract toxicity in normal cells

To assess the toxicity of the plant extracts on primary cells (peritoneal murine macrophages), the MTT toxicity assay was used. Thioglycollate-elicited mouse peritoneal macrophages were harvested from female BALB/C mice [34]. Experimental protocol was again undertaken in accordance with the ethical guidelines and the permission of the University of Hyderabad, School of Life Sciences’ animal ethics committee was obtained. Toxicity toward mouse peritoneal macrophages was assessed with cells plated in 96-well plates at a density of 2 × 10^6^ cells per well (in 200 μL volume). After adherence, the medium was removed and replaced by one of the media containing the plant extracts. The plates were incubated for 72 hrs at 37°C in a humidified 5% CO_2_ incubator. Control cells were incubated with culture medium alone. Cell viability was determined by a colorimetric assay with the tetrazolium salt MTT [33]. Absorbance of the formed formazan product was measured at a test wavelength of 540 nm. Results were expressed as percentage cellular viability of the extracts.

#### Calculations and statistical analysis

The percentage inhibitions of radicals, lipid peroxidation and cytotoxic activities of the extracts were calculated using the formula:

(1) $$\text{Percentage}\;\text{inhibition}=\left(A_{\text{control}}-A_{\text{sample}}\right)/A_{\text{control}}\times 100$$

All results are expressed as mean ± standard deviation (SD) values average from 3 to 4 independent experiments performed in duplicate. IC_*50*_ value (the concentration of the extracts required to scavenge 50% of radicals) was calculated for different extracts of *A. precatorius.*

Statistical differences between correlated samples were evaluated using Student’s *t*-test and composite treatments were compared using one-way analysis of variances (ANOVA) and considered significantly different where probability values were found to be equal to or less than 0.05. Statistical tests as well as mean and SD calculations and graphical representation of the results were performed using GraphPad Prism v5 and Sigmaplot v11.0 software’s.

## Results and discussion

### Determination of phyto-constituents

The results of total phenolic content in leaf extracts of *A. precatorius* evaluated using Folin-Ciocalteu method are presented in Table 1. Total polyphenolic content in different extracts varied with the solvent used in this study. The results indicated that water extract (APW) possessed an abundance of phenolics amounting to 25.48 ± 0.62 mg GAE/g dw, while GAE value of ethyl acetate (APE) extract was 23.57 ± 0.31 mg/g dw. Ethanol extract (APA) and hexane extract (APH) showed less amount of GAE at 7.44 ± 0.10 mg/g dw and 1.65 ± 0.22 mg/g dw respectively. It suggested that major phenolics of *A. precatorius* were mainly located in water extract. The extracts of *A. precatorius* also contained significant amount of flavonoids (Table 1). The flavonoid contents of extracts in terms of quercetin equivalents were between 6.20 ± 0.41 and 17.16 ± 1.04 mg/g dw. The flavonoid contents in APE (17.16 ± 1.04 mg/g dw) was higher compared to other three extracts. The plant derived antioxidants especially polyphenols and flavonoids have recently attracted medicinal attention as bioactive agents with anticancer, antidiabetic, antimicrobial, hepatoprotective, neuroprotective and cardioprotective properties [35-37]. They have been ascribed to have resilient antioxidant activity and help in protecting cells against oxidative damage caused by free radicals due to their redox properties, which enable them to act as reducing agents, hydrogen donors and singlet oxygen quenchers [38-41].

**Table 1:** **Quantitative estimation of phytochemicals and antioxidant activities of different extracts of *A. precatorius***

**Type of Extract**	**Total Phenols**^**a**^	**Total Flavonoid**^**b**^	**Total antioxidant capacity**^**c**^	**Ferric reducing antioxidant power**^**c**^
**APH**	1.65 ± 0.22	6.20 ± 0.41	7.16 ± 0.43	2.67 ± 0.40
**APE**	23.57 ± 0.31	17.16 ± 1.04	17.92 ± 0.38	8.45 ± 0.65
**APA**	7.44 ± 0.10	7.23 ± 0.68	24.73 ± 0.72	10.28 ± 0.47
**APW**	25.48 ± 0.62	10.70 ± 0.56	16.66 ± 0.68	13.34 ± 0.35

a: Gallic acid; b: Quercetin; c: Ascrobic acid equivalents mg/g dw plant material respectively. Each value is expressed as a mean ± standard deviation (n = 3).

### Antioxidant ability assays

#### Phosphomolybdenum assay

In phosphomolybdenum assay, which is a quantitative method to evaluate the antioxidant capacity, all the extracts exhibited different degrees of activity as shown in Table 1. The reducing power of a compound is associated with electron donating capacity and serves as an indicator of antioxidant activity [42,43]. Results indicated that APA has higher antioxidant capacity (24.73 ± 0.72 mg ascorbic acid equivalent/g dw) than other three extracts which showed antioxidant capacity in the order: APE (17.92 ± 0.38 mg AAE/g dw) > APW (16.66 ± 0.68 mg AAE/g dw) > APH (7.16 ± 0.43 mg AAE/g dw).

#### Ferric-reducing/antioxidant power (FRAP) assay

In reducing power assay, the presence of reductants (antioxidants) in samples would result in the reduction of Fe^3+^ to Fe^2+^ by donating an electron which serves as a significant reflection of antioxidant activity [44]. The amount of Fe^2+^ complex can be monitored by measuring the formation of Perl’s Prussian blue at 700 nm. Increasing absorbance at 700 nm indicates an increase in reductive ability [45]. Earlier reports suggest that some phenolic compounds such as flavonoids and phenolic acids exhibited antioxidant activity through their reductive capacity in a Fe^3+^- Fe^2+^ system [46]. All the four extracts showed some degree of electron donating capacity and reduced Fe^3+^ to Fe^2+^. The reducing ability of the extracts was in range of 13.34 ± 0.35 to 2.67 ± 0.40 AAE mg/g dw (Table 1). The FRAP values for APW was found to be higher than other three extracts. This suggests that APW has a significant ability to react with free radicals to alter them into more stable non-reactive species and to terminate radical chain reaction.

#### DPPH^•^ radical scavenging activity

**Figure 1:**
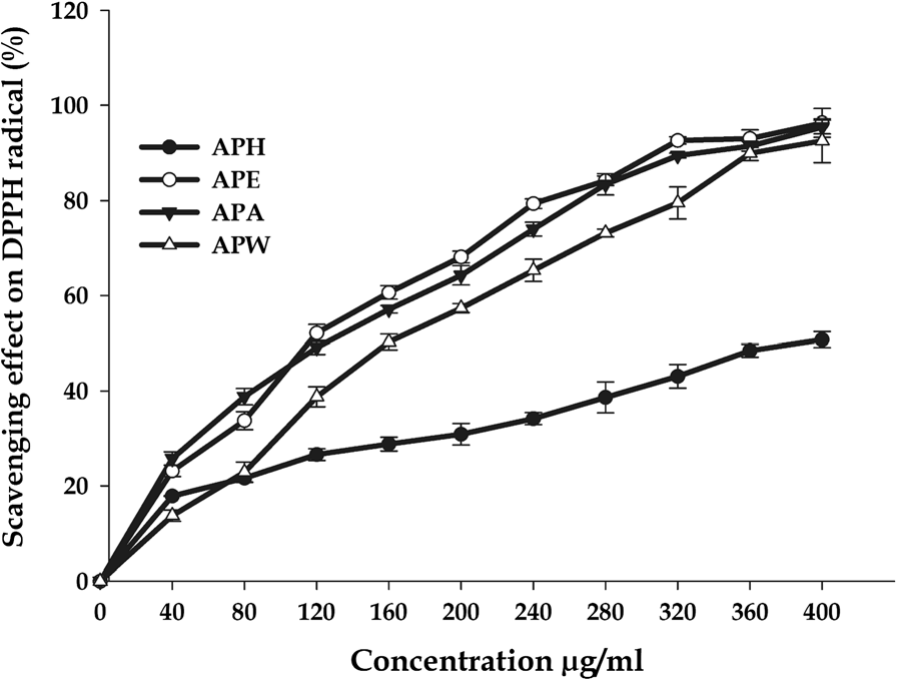
**DPPH radical-scavenging activities of *A. precatorius *leaf extracts at different concentrations.**

DPPH assay provides basic information on antiradical activity of extracts and its results can indicate the presence of phenolic and flavonoid compounds in plant extracts [47]. Very significant antioxidant activities were found in all the four extracts and positive control, which increased with increasing concentration (Figure 1). DPPH activity values for APE, APA and APW were found to be nearer to each other. The APE and APW were able to inhibit the formation of DPPH^•^ radicals with a percentage inhibition of 96.35 ± 2.98 and 92.63 ± 4.63% respectively at the highest concentration of 400 μg/ml with the IC_*50*_ values of 57.66 ± 1.32 and 79.97 ± 1.84 μg/mL respectively (Table 1). Previous reports have demonstrated that ethyl acetate fractions are good sources of antioxidant compounds [48,49]. APA also exhibited potent DPPH scavenging activity (95.14 ± 3.44%) in this study and its IC_*50*_ (60.67 ± 1.03 μg/mL) was near to that of APE, in which phenolic levels were approximately 3.5 times higher. Several groups have pronounced a positive correlation between phenolic content and antioxidant activity [50-52] using similar assay systems, but our study could not establish correlation in similar manner. It could be due to the presence of other reducing compounds that probably interfere with the Folin-Ciocalteu assay and/or the presence of other non-phenolic compounds with antioxidant effects. APH showed less inhibitory action as compared to other extracts with the percentage inhibition of 50.84 ± 5.68 at same concentration with the IC_*50*_ value 196.70 ± 1.84 μg/mL. The IC_*50*_ values of ascorbic acid and quercetin (positive controls) were found to be 3.80 ± 0.43 and 9.84 ± 0.6 μg/mL respectively. This behaviour can be explained by different composition of each extracts as there are compounds that react quickly with DPPH and others that have a slower reaction mechanism and required extremely high concentration to have a significant effect [53].

#### Hydroxyl radical scavenging activity

**Figure 2:**
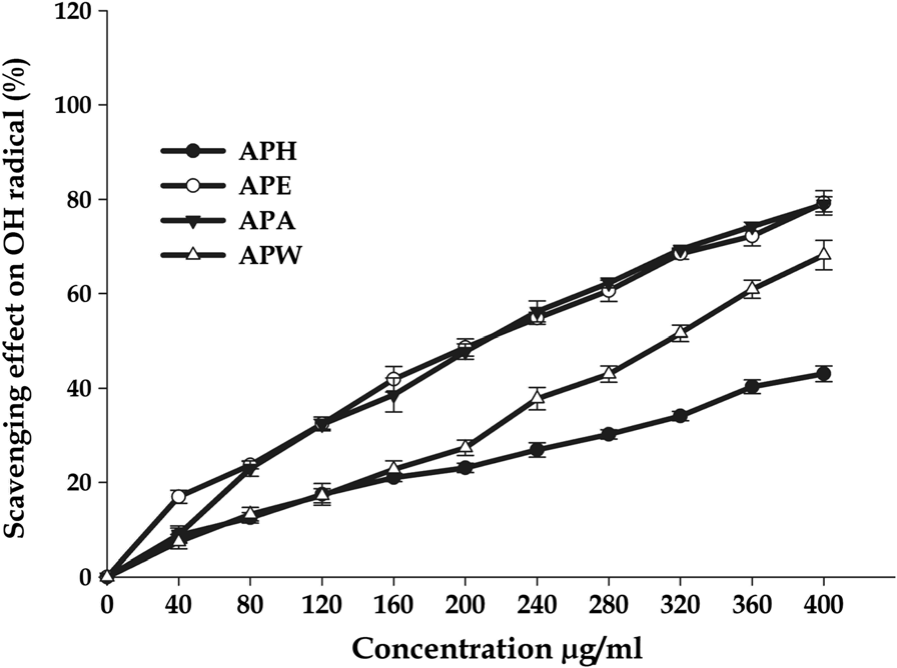
**Hydroxyl radical scavenging activities of *A. precatorius *leaf extracts at different concentrations.**

The hydroxyl radical is known to be the most reactive oxygen radical and it severely damages neighbouring bio-molecules in the body, such as protein and DNA, resulting into mutagenesis, carcinogenesis and cytotoxicity [54,55]. Therefore, removal of hydroxyl radical is possibly one of the most effective defences of a living body against various diseases. A significant decrease in concentration of hydroxyl radical was observed due to *A. precatorius* extracts (Figure 2; Table 2). All the extracts exhibited significant activity, above 40% in a concentration - dependent manner with maximal inhibition of 79.52 ± 2.57% at 400 μg/mL by APE with IC_*50*_ value of 205.51 ± 3.08 μg/mL. APA (78.97 ± 1.60%; IC_*50*_ = 209.33 ± 4.13 μg/mL) and APW (68.18 ± 3.14%; IC_*50*_ = 309.90 ± 5.21 μg/mL) extracts were also found to be significant powerful quenchers of ^•^OH radical as compared to ascorbic acid (IC_*50*_ = 62.40 ± 3.72). APH was found to be weak scavenger of ^•^OH with the *IC*_*50*_ value of 464.25 ± 4.43 μg/mL. Our results suggested that hydroxyl radical scavenging ability of extracts of *A. precatorius* are comparable to or even higher than earlier published reports [12,56,57] and could help in preventing oxidative damage in the human body.

**Table 2:** **IC**_***50 ***_**values obtained in the antioxidant activity assays**

**Sample**	**IC**_***50 ***_**μg/mL**					
	**DPPH**	**OH**	**H**_**2**_**O**_**2**_	**NO**	**O**_**2**_^**-·**^	**Lipid peroxidation**
**APH**	196.70 ± 2.06	464.25 ± 4.43	112.59 ± 3.24	192.45 ± 3.76	427.26 ± 5.72	377.07 ± 5.23
**APE**	57.66 ± 1.32	205.51 ± 3.08	121.02 ± 3.14	107.58 ± 2.12	143.44 ± 3.28	45.46 ± 3.71
**APA**	60.67 ± 1.03	209.33 ± 4.13	92.83 ± 3.23	145.96 ± 2.17	157.07 ± 2.56	285.22 ± 4.63
**APW**	79.97 ± 1.84	309.90 ± 5.21	152.35 ± 1.06	264.95 ± 4.24	201.45 ± 6.23	302.02 ± 4.11
**Ascorbic acid**	6.86 ± 0.92	62.40 ± 3.72	--	19.90 ± 2.30	32.86 ± 3.78	48.72 ± 3.20
**Quercetin**	14.34 ± 1.64	--	245.30 ± 4.60	21.09 ± 2.16	--	--

Each value is expressed as a mean ± standard deviation (n = 3).

#### Hydrogen peroxide scavenging assay

**Figure 3:**
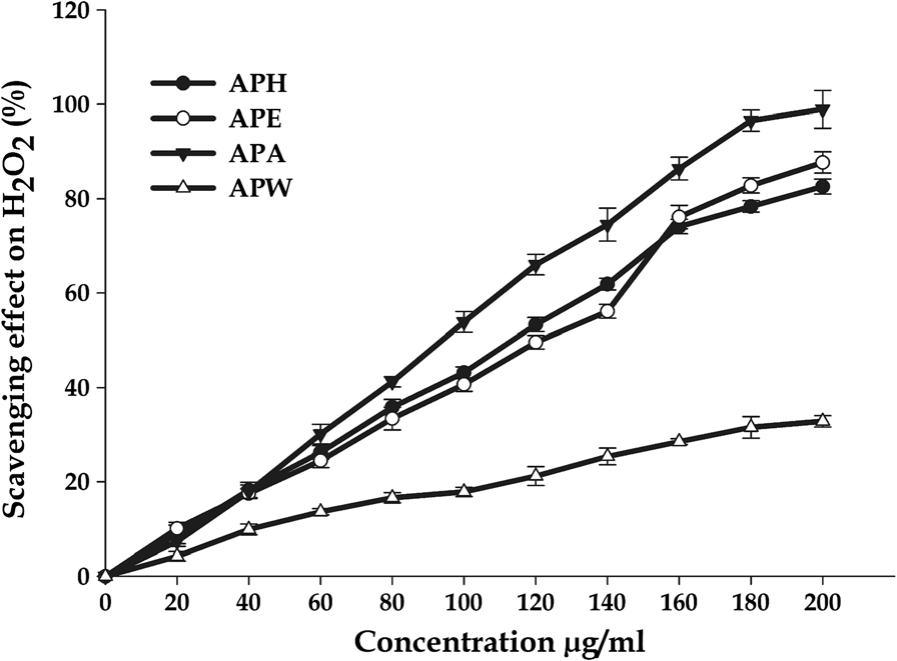
**Hydrogen peroxide scavenging activities of *A. precatorius *leaf extracts at different concentrations.**

Hydrogen peroxide (H_2_O_2_) is a biologically relevant, non-radical reactive oxygen species and is inevitably generated as a by-product of normal aerobic metabolism. However, when concentration increases under stress conditions, H_2_O_2_ could be detrimental for cells [58] and, furthermore, could be converted into other ROS such as hydroxyl radicals. Thus, H_2_O_2_ scavenging activity becomes a crucial characteristic of total antioxidant activity. In this study, APA extract (IC_*50*_ = 92.83 ± 3.23 μg/ml) was found to be efficient scavenger of hydrogen peroxide radical, while APW extract (IC_*50*_ = 152.35 ± 1.06 μg/mL) was least efficient. The APH and APE extracts also exhibited comparable efficiency with IC_*50*_ = 112.59 ± 3.24 and 121.02 ± 3.14 μg/mL respectively (Figure 3; Table 2). The H_2_O_2_ scavenging capacity of all extracts was also better than that of quercetin tested in the same conditions. The results strongly suggest that these extracts contain the necessary compounds for radical elimination. Many reports have already proven that nutritive phenols play a significant role in protecting mammalian and bacterial cells from cytotoxicity induced by H_2_O_2_[59-61], indicating that the observed activity of plants extracts could be due to the presence of phenols.

#### Nitric oxide scavenging activity

**Figure 4:**
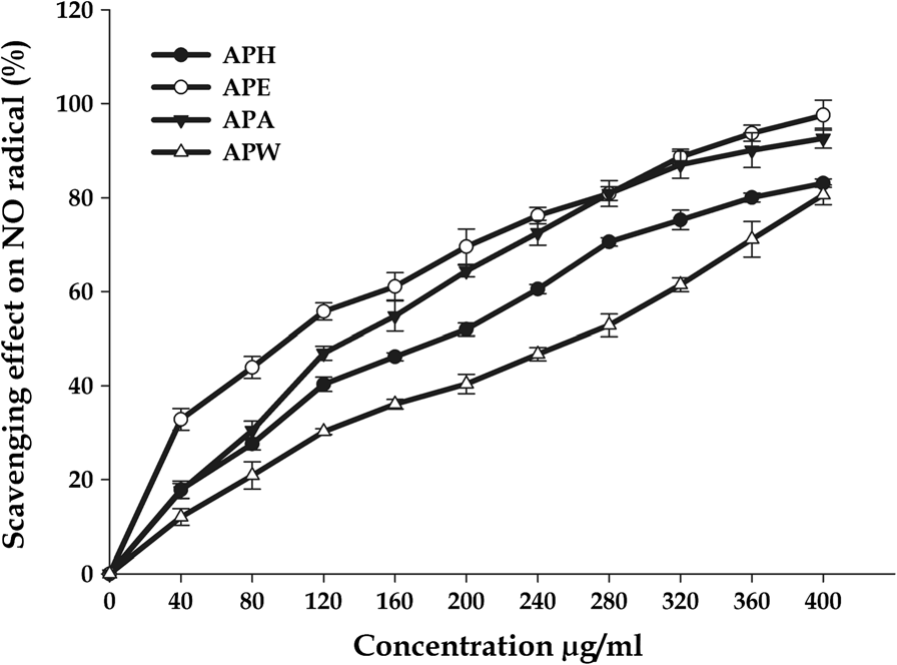
**Nitric oxide scavenging activities of *A. precatorius *leaf extracts at different concentrations.**

Initially NO was regarded to have only beneficial effects, but it has been found that over production of NO is closely associated with different pathological diseases, such as chronic inflammation, autoimmune diseases and cancer [62]. The NO radicals play an important role in inducing inflammatory response and their toxicity multiplies only when they react with O_2_^.-^ radicals to form peroxynitrite which damages the biomolecules such as proteins, lipids and nucleic acids, and therefore injures the host tissue [56]. The measure of NO radical scavenging activity was based on the principle that sodium nitroprusside in an aqueous solution at physiological pH spontaneously generates nitric oxide, which interacts with oxygen to produce nitrite ions that can be estimated using a Griess reagent. Scavengers of nitric oxide act against oxygen, leading to reduced production of nitrite ions which can be monitored at 546 nm. *A. precatorius* extracts showed significant decrease in NO radical due to the scavenging ability of extracts. All the extracts exhibited significant NO scavenging activity in a concentration dependent manner (Figure 4). The results clearly identify APE as better NO scavenger where percentage inhibition reached to 97.58 ± 3.12% with an IC_*50*_ value of 107.58 ± 2.12 μg/mL followed by APA whose inhibition of generation of NO reached up to 92.70 ± 2.13 (IC_*50*_ = 145.96 ± 2.17 μg/mL) in a concentration - dependent manner and a gradual decline thereafter at the higher concentrations. The APH and APW extracts were also efficient scavengers of NO (83.11 ± 0.89% and 80.62 ± 2.11%) with slightly higher IC_*50*_ Values, 192.45 ± 3.76 and 264.95 ± 4.24 μg/mL respectively.

#### Superoxide radicals scavenging activity

**Figure 5:**
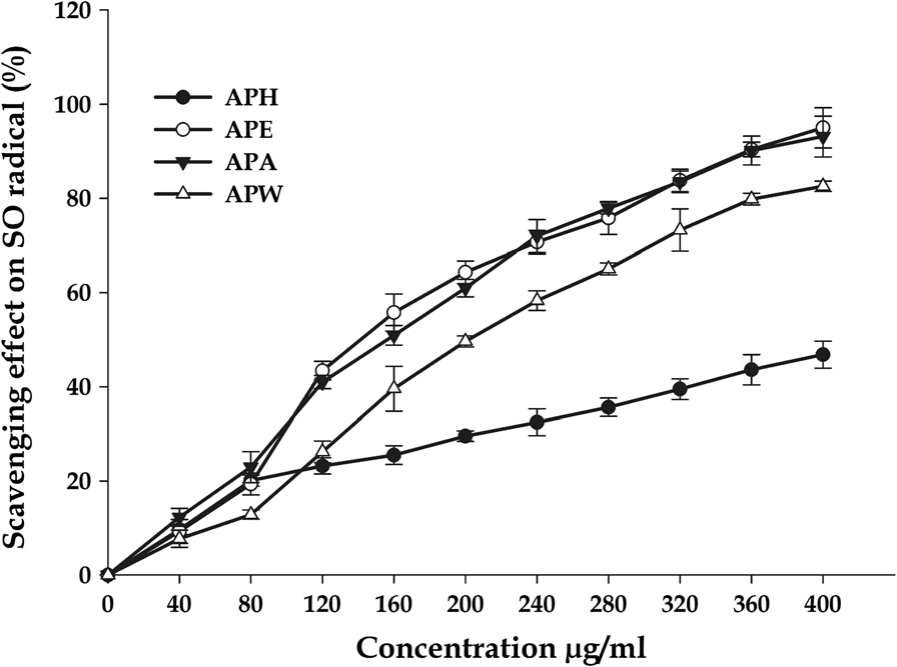
**Superoxide scavenging activities of different extracts of *A. precatorius *at different concentrations.**

Superoxide anion (O_2_^.-^) is one of the most important representatives of free radicals. It acts as a precursor of more reactive oxidative species such as single oxygen and hydroxyl radicals that have the potential of reacting with biological macromolecules and thereby inducing tissue damage, and plays a vital role in peroxidation of lipids [63-65]. In the present study, the inhibitory effect of *A. precatorius* extracts on superoxide radicals was in a concentration dependent manner (Figure 5). High inhibitions were observed at very low extract concentrations. At 400 μg/mL of tested extract, the scavenging effects on superoxide radical were found to be 95.01 ± 4.29%; IC_*50*_ value = 143.44 ± 3.28 μg/mL for APE and 93.15 ± 4.36%; IC_*50*_ value = 157.07 ± 2.56 μg/mL for APA. Moreover, APW and APH also possess the significant scavenging effect 73.25 ± 4.50%; IC_*50*_ value = 201.45 ± 6.23 μg/mL and 46.81 ± 2.87%; IC_*50*_ value = 427.26 ± 5.72 μg/mL respectively, however with higher IC_*50*_ values.

#### Inhibition of lipid peroxidation assay

**Figure 6:**
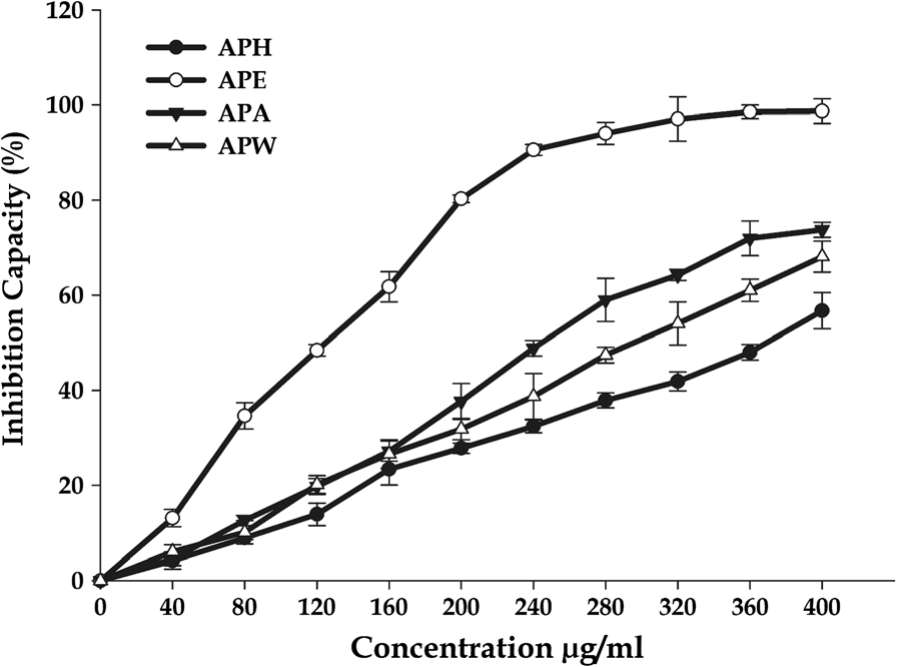
**Effects of different concentrations of crude extracts from the leaf of *A. precatorius *on Fe (II) induced TBARS production in liver.**

Lipid peroxidation involves the formation and propagation of lipid radicals with numerous deleterious effects, including destruction of membrane lipids, metabolic disorders and inflammation. Production of malondialdehyde (MDA) is a hallmark of this process. This process is initiated by hydroxyl and superoxide radicals leading to the formation of peroxy radicals (LOO^.^) that ultimately propagates chain reaction in lipids. Thus, antioxidants which are capable of scavenging peroxy radicals could prevent lipid peroxidation. In this study, we measured the potential of *A. precatorius* extracts to inhibit lipid peroxidation in rat liver microsomes, induced by the Fe^2+^/ascorbate system (Figure 6). Different extracts protected against lipid peroxidation induced by Fe^2+^, considerably reduced MDA content in a concentration-dependent manner. APE had the greatest inhibiting activity (98.70 ± 2.56%); with the lowest IC_*50*_ value 45.46 ± 3.71 μg/mL. When compared to the activity of standard, (ascorbic acid, IC_50_ = 48.72 ± 4.23 μg/mL), inhibiting activity against lipid peroxidation of APE was very high considering that the extract was a mixture of a great number of components against pure compound used as standards. The other extracts (APA, APW and APH) proved to be much weaker inhibitors of lipid peroxidation than APE with the percentage inhibition of 73.75 ± 1.60%, 68.09 ± 3.26% and 56.73 ± 3.81% respectively with higher IC_*50*_ values than that of APE (Table 2).

#### Antiproliferative activity

**Figure 7:**
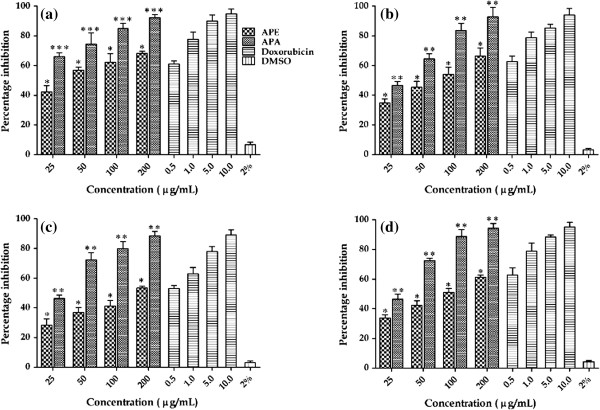
**Antiproliferative activity of *A. precatorius *leaf extracts (APA and APE) against: (a) COLO-205 (b) Y79 (c) HepG2 and (d) SupT1 cell lines. **Significant p value (*** p < 0.001, ** p < 0.01 and * p <0.05 were obtained by Student’s *t *test analysis. Composite treatments were compared using one-way analysis of variances (ANOVA) and probability values were found to be equal to or less than 0.05 for all the four cell lines.

Deregulation of cell proliferation, together with suppressed apoptosis, is a minimal, common platform for all cancer evolution and progression [66]. Uncontrolled cell division is the primary key in the progression of cancer tumors. In order to evaluate *A. precatorius* as a potential therapy for cancer, different extracts were assayed against a panel of four human cancer cell lines: Colo-205, Y79, HepG2 and SupT1. The antiproliferative effects were quantified in terms of cytotoxicity (percentage inhibition) and IC_*50*_ values were also determined with lower IC_*50*_ values indicating a higher antiproliferative activity. Out of four extracts tested, only APA and APE demonstrated significantly effective antiproliferative activities in a concentration dependent manner, whereas APH and APW extracts did not inhibit the proliferation of tumor cells, thus indicating their non - cytotoxic properties. In fact, APA was by far the strongest inhibitor of tumor cell proliferation with above 85% growth inhibition of all tested cell lines, while as APE was slightly weaker inhibitor of growth of cell lines than APA (Figure 7a-7d; Table 3). APA exerted the highest cytotoxicity at a concentration of 200 μg/mL against Colo-205 (92.25 ± 2.05%) and Y79 (92.80 ± 6.34%) cells with an IC_*50*_ value of 18.91 ± 1.06 and 26.74 ± 1.34 μg/mL respectively. Whereas APE inhibits growth up to 68.33 ± 1.41% and 66.40 ± 5.44% against Colo-205 and Y79 cells respectively at the same concentration of 200 μg/mL with higher IC_50_ values of 29.57 ± 2.02 and 35.94 ± 2.10 μg/mL respectively. In addition, APA also showed significant inhibition activity on other two human cancer cell lines HepG2 (88.52 ± 3.04%) and SupT1 (94.12 ± 3.34%) at 200 μg/mL with the IC_*50*_ value 27.03 ± 1.03 and 26.89 ± 3.24 μg/mL respectively. The APE showed moderate ability to inhibit cancer cell growth in a concentration-dependent manner with IC_*50*_ value of 44.31 ± 3.07 μg/ml for HepG2 and 37.00 ± 2.38 μg/ml for SupT1.

**Table 3:** **Percentage inhibition of cancer cell proliferation and IC**_***50 ***_**values**

**Sample**	**Type of cell line**							
	**Colo – 205**		**Y79**		**HepG2**		**SupT1**	
	**%age Inhibition**	**IC**_***50 ***_**Value**	**% age Inhibition**	**IC**_***50 ***_**Value**	**% age Inhibition**	**IC**_***50 ***_**Value**	**% age Inhibition**	**IC**_***50 ***_**Value**
**APE **(200 μg/mL)	68.33 ± 1.41^*^	29.57 ± 2.02	66.40 ± 5.44^*^	35.94 ± 2.10	53.33 ± 1.21^*^	44.31 ± 3.07	61.34 ± 1.32^*^	37.00 ± 2.38
**APA **(200 μg/mL)	92.25 ± 2.05^***^	18.91 ± 1.06	92.80 ± 6.34^*^	26.74 ± 1.34	88.52 ± 3.04^**^	27.03 ± 1.03	94.12 ± 3.34^**^	26.89 ± 3.24
**Doxorubicin **(Standard) 10 μg/mL	94.81 ± 3.42	0.41 ± 0.08	94.20 ± 4.11	0.39 ± 0.10	89.18 ± 3.42	0.47 ± 0.08	95.20 ± 3.11	0.39 ± 0.11
**DMSO 2% **(Solvent control)	6.67 ± 1.67	--	3.33 ± 0.78	--	3.13 ± 1.01	--	4.33 ± 0.78	--

Values were the means of four replicates ± standard deviation (SD). Significant p value (*** p < 0.001, ** p < 0.01 and * p <0.05 were obtained by Student’s *t *test analysis. Composite treatments were compared using one-way analysis of variances (ANOVA) and probability values were found to be equal to or less than 0.05 for all the four cell lines.

The criterion for cytotoxicity for the crude extracts, as established by the National Cancer Institute (NCI), is an IC_*50*_ value lower than 30 μg/mL [67]. In this study, the APA crude extract displays an IC_*50*_ values less than 30 μg/mL in all the four tested cell lines, which established APA as more active extract than APE. Comparatively, Doxorubicin, an anticancer drug used in this study as a positive control, demonstrated IC_*50*_ values in the tumor cell lines ranging from 0.39-0.47 μg/mL. Although, the activity of APA and APE are weak in comparison to the standard drug, which could be due to the crude nature of the extracts and can be further enhanced by the purification.

#### Assessment of drug toxicity

**Figure 8:**
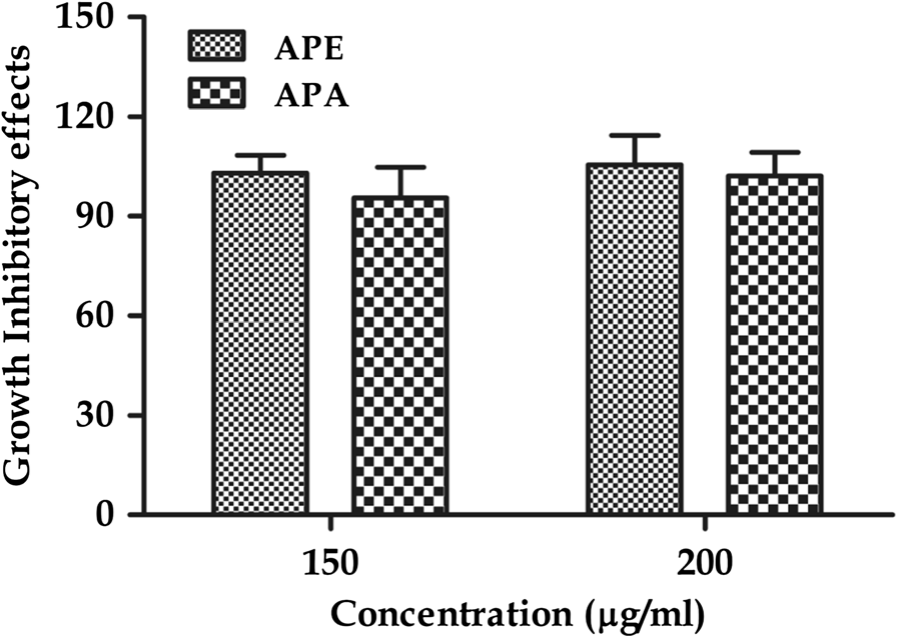
**Growth inhibitory effects of *A. precatorius *leaf extract (APA and APE) on peritoneal macrophages. **Cell viability was determined by MTT assay as described in material and methods section.

The cytotoxic effect of APA and APE was studied in murine peritoneal macrophages using the MTT method. The results clearly indicated that plant extracts were virtually nontoxic and had no inhibitory effect on cell proliferation in peritoneal macrophages and there was minimal reduction in cell survivability (Figure 8). The percentage viability was above 90% at the highest concentration of 200 μg/mL. This suggests that APA and APE extracts did not show any kind of toxic effect on the normal cells. Therefore, the cytotoxicity of the active extracts was found to be highly selective against the cancer cell lines used.

During the past decade, both *in vivo* and *in vitro* studies have suggested that natural antioxidants such as phenolics, carotenoids, tocotrienols exhibit antitumor activities by inhibiting the growth and proliferation of many cancer cells such as breast, lung and liver cancer cells [68-71]. These observations and reports (with regard to the cytotoxicity of the plant extracts) indicate that there are great differences among the antiproliferative activity of the same plant species, depending on plant parts and extraction solvents used. Furthermore, the different cell lines vary in their sensitivity to the same plant extract.

## Conclusion

This work has gathered experimental evidence that *A. precatorius* leaf extracts contained substantial amount of polyphenols and flavonoids and exhibited significant antioxidant activity by effectively scavenging various free radicals. Additionally, it has been demonstrated that the *A. precatorius* leaf extracts (APA and APE) are potential antiproliferative agents without any toxic effect on normal cells. The antioxidant and antiproliferative activities might be due to the synergistic actions of bioactive compounds present in them. Therefore, the plant has promising compounds to be tested as potential antioxidant drugs for treatment of diseases resulting from oxidative stress. However, these findings warrant extensive studies on chemical profiles and mechanistic action of antiproliferative and antioxidant activities. The study will be helpful to understand this important herbal medicine and further studies are underway in our laboratory.

## Competing interests

The authors declare that they have no competing interests.

## Authors’ contributions

MZG conceived the study, carried out all the experimentation, acquisition and analysis of data and drafting of the manuscript. FA was involved in cell culturing, MTT assay. AKK provided technical support and advice in cytotoxic studies. IAQ helped in drafting and revision of manuscript. IAG conceived, designed, supervised the study and revised the manuscript. All authors have read and approved the final manuscript.

## Pre-publication history

The pre-publication history for this paper can be accessed here:

http://www.biomedcentral.com/1472-6882/13/53/prepub
